# Supplementary material for: Capturing Multiscale Dynamics of Aortic Valve Calcification with a Coupled Fluid−Structure and Systems Biology Model
Source: ACS Omega. 2026 Mar 30;11(14):21974–88. doi: 10.1021/acsomega.5c12886 (PMC13084384; doi:10.1021/acsomega.5c12886)
Supplement: Supplementary file 1 [file ao5c12886_si_001.pdf]

Supplementary Material for:

Capturing Multi-Scale Dynamics of Aortic Valve Calcification With a Coupled Fluid–Structure and Systems Biology Model

Michael Quan[1], Tianyou Xie[1], Leonard A. Harris[2,3], and Haoxiang Luo*[1]

[1]*Department of Mechanical Engineering, Vanderbilt University, Nashville, TN*
[2]*Department of Biomedical Engineering, University of Arkansas, Fayetteville, AR*
[3]*Cancer Biology Program, Winthrop P. Rockefeller Cancer Institute, Little Rock, AR*

January 29, 2026

*Corresponding author: haoxiang.luo@vanderbilt.edu

Supporting Information

Species in the integrated SB aortic calcification model

Table S1: *List of species*

Species	Description
$ALK5_c$	Cytoplasmic ALK5 (TGFβ receptor type I)
$ALK5_c\text{-}R2_c\text{-}TGF\beta_{\mathrm{act}}$	Cytoplasmic ALK5–R2–TGFβ_{act} receptor complex
$ALK5_s$	Surface ALK5 (TGFβ receptor type I)
$ALK5_s\text{-}R2_s\text{-}TGF\beta_{\mathrm{act}}$	Surface ALK5–R2–TGFβ_{act} receptor complex
Ca	Intracellular calcium produced downstream of SMAD-dependent transcription
FoamCell	Foam cell (lipid-loaded macrophage)
sGC	Soluble guanylyl cyclase
sGC-GTP	sGC–GTP complex
GMP	Guanosine monophosphate produced by PDE hydrolysis of cGMP
cGMP	Cyclic GMP, second messenger produced by sGC
GTP	Guanosine triphosphate, substrate for sGC catalytic activity
LDL	Low-density lipoprotein
oxLDL	Oxidized LDL
Macrophage	Differentiated monocyte involved in inflammation and TGFβ activation
Monocyte	Circulating immune cell that differentiates into macrophages
NO	Nitric oxide
NO-sGC	NO–sGC complex
NO-sGC-GTP	NO–sGC–GTP complex
PDE	Phosphodiesterase enzyme
PDE-cGMP	PDE–cGMP complex
PKG	Inactive protein kinase G
PKG_{act}	Active PKG (activated by cGMP)
$R2_c$	Cytoplasmic TGFβ receptor type II
$R2_s$	Surface TGFβ receptor type II
$R2_s\text{-}TGF\beta_{\mathrm{act}}$	Surface R2–active TGFβ complex
$SMAD2_c$	Cytoplasmic SMAD2
$pSMAD2_c$	Cytoplasmic phosphorylated SMAD2
$SMAD2_c\text{-}ALK5_c\text{-}R2_c\text{-}TGF\beta_{\mathrm{act}}$	Cytoplasmic TGFβ receptor complex with SMAD2 and active TGF-β ligand
$pSMAD2_c\text{-}SMAD4_c$	Cytoplasmic pSMAD2–SMAD4 heterodimer
$SMAD2_n$	Nuclear SMAD2
$pSMAD2_n$	Nuclear phosphorylated SMAD2
$SMAD2_n\text{-}SMAD4_n$	Nuclear SMAD2–SMAD4 heterodimer
$pSMAD2_n\text{-}SMAD4_n$	Nuclear pSMAD2–SMAD4 heterodimer
$SMAD3_c$	Cytoplasmic SMAD3
$pSMAD3_c$	Cytoplasmic phosphorylated SMAD3
$pSMAD3_{c,\mathrm{inh}}$	Inhibited cytoplasmic SMAD3
$SMAD3_c\text{-}ALK5_c\text{-}R2_c\text{-}TGF\beta_{\mathrm{act}}$	Cytoplasmic TGFβ receptor complex with SMAD3 and active TGF-β ligand
$pSMAD3_c\text{-}SMAD4_c$	Cytoplasmic pSMAD3–SMAD4 heterodimer
$SMAD3_n$	Nuclear SMAD3
$pSMAD3_n$	Nuclear phosphorylated SMAD3
$SMAD3_n\text{-}SMAD4_n$	Nuclear SMAD3–SMAD4 heterodimer
$pSMAD3_n\text{-}SMAD4_n$	Nuclear pSMAD3–SMAD4 heterodimer
$SMAD4_c$	Cytoplasmic SMAD4
$SMAD4_n$	Nuclear SMAD4

Table S1 (*cont.*)

Species	Description
$TGF\beta$	Latent (inactive) TGF-β
$TGF\beta_{\text{act}}$	Active TGF-β

Reactions for the three sub-models of the SB aortic calcification model

Table S2: *Inflammation model*

Reaction	Description
$\emptyset \xrightarrow{k_{\text{inf1}}} LDL$	LDL penetrates the endothelium and enters the leaflets
$LDL \xrightarrow{k_{\text{inf2}}} \emptyset$	LDL exits the leaflets through the endothelium
$LDL \xrightarrow{k_{\text{inf3}}} oxLDL$	LDL oxidation
$oxLDL + Macrophage \xrightarrow{k_{\text{inf4}}} FoamCell$	Macrophages absorb oxLDL to become lipid-laden foam cells
$oxLDL \xrightarrow{k_{\text{inf5}}} oxLDL + Monocyte$	Oxidized LDL promotes monocyte capture by increasing endothelial adhesion molecule expression, enabling circulating monocytes to enter the valve leaflets
$Monocyte \xrightarrow{k_{\text{inf6}}} Macrophage$	Monocytes differentiate into macrophages
$Monocyte \xrightarrow{k_{\text{inf7}}} \emptyset$	Monocyte apoptosis
$Macrophage + TGF\beta \xrightarrow{k_{\text{inf8}}} Macrophage + TGF\beta_{\text{act}}$	Macrophages activate latent TGF-β
$FoamCell + TGF\beta \xrightarrow{k_{\text{inf8}}} FoamCell + TGF\beta_{\text{act}}$	Foam cells activate latent TGF-β
$FoamCell \xrightarrow{k_{\text{inf9}}} \emptyset$	Foam cell apoptosis
$\emptyset \xrightarrow{k_{\text{inf10}}} TGF\beta$	TGF-β secretion by macrophages and foam cells; the rate saturates at high cell numbers
$TGF\beta_{\text{act}} \xrightarrow{k_{\text{inf11}}} \emptyset$	Activated TGF-β degrades
$TGF\beta \xrightarrow{k_{\text{inf12}}} \emptyset$	Latent TGF-β degrades
$Macrophage \xrightarrow{k_{\text{inf13}}} \emptyset$	Macrophage apoptosis

Table S3: *SMAD signaling model*

Reaction	Description
$TGF\beta_{\text{act}} + R2_s \underset{k_{\text{sma1b}}}{\overset{k_{\text{sma1a}}}{\rightleftharpoons}} R2_s\text{-}TGF\beta_{\text{act}}$	Activated TGF-β binds and unbinds from TGFβRII at the VIC surface
$R2_s\text{-}TGF\beta_{\text{act}} + ALK5_s \underset{k_{\text{sma2b}}}{\overset{k_{\text{sma2a}}}{\rightleftharpoons}} ALK5_s\text{-}R2_s\text{-}TGF\beta_{\text{act}}$	The TGFβ–TGFβRII complex binds and unbinds from ALK5 (TGFβRI)
$ALK5_s\text{-}R2_s\text{-}TGF\beta_{\text{act}} \overset{k_{\text{sma3}}}{\rightleftharpoons} ALK5_c\text{-}R2_c\text{-}TGF\beta_{\text{act}}$	The receptor complex moves between the cell surface and cytoplasm
$ALK5_c\text{-}R2_c\text{-}TGF\beta_{\text{act}} + SMAD2_c \underset{k_{\text{sma4b}}}{\overset{k_{\text{sma4a}}}{\rightleftharpoons}} SMAD2_c\text{-}ALK5_c\text{-}R2_c\text{-}TGF\beta_{\text{act}}$	The receptor complex binds and unbinds from SMAD2
$ALK5_c\text{-}R2_c\text{-}TGF\beta_{\text{act}} + SMAD3_c \underset{k_{\text{sma4b}}}{\overset{k_{\text{sma4a}}}{\rightleftharpoons}} SMAD3_c\text{-}ALK5_c\text{-}R2_c\text{-}TGF\beta_{\text{act}}$	The receptor complex binds and unbinds from SMAD3
$SMAD2_c\text{-}ALK5_c\text{-}R2_c\text{-}TGF\beta_{\text{act}} \xrightarrow{k_{\text{sma5}}} ALK5_c\text{-}R2_c\text{-}TGF\beta_{\text{act}} + pSMAD2_c$	Bound SMAD2 is phosphorylated by the receptor complex

Table S3 (*cont.*)

Reaction	Description
$SMAD3_c\text{-}ALK5_c\text{-}R2_c\text{-}TGF\beta_{\mathrm{act}} \xrightarrow{k_{\mathrm{sma5}}} ALK5_c\text{-}R2_c\text{-}TGF\beta_{\mathrm{act}} + pSMAD3_c$	Bound SMAD3 is phosphorylated by the receptor complex
$pSMAD2_c + SMAD4_c \underset{k_{\mathrm{sma6b}}}{\overset{k_{sma6a}}{\rightleftharpoons}} pSMAD2_c\text{-}SMAD4_c$	pSMAD2 heterodimerizes with SMAD4
$pSMAD3_c + SMAD4_c \underset{k_{\mathrm{sma6b}}}{\overset{k_{sma6a}}{\rightleftharpoons}} pSMAD3_c\text{-}SMAD4_c$	pSMAD3 heterodimerizes with SMAD4
$pSMAD2_c\text{-}SMAD4_c \xrightarrow{k_{\mathrm{sma7}}} pSMAD2_n\text{-}SMAD4_n$	pSMAD2–SMAD4 heterodimer translocates to the nucleus
$pSMAD3_c\text{-}SMAD4_c \xrightarrow{k_{\mathrm{sma7}}} pSMAD3_n\text{-}SMAD4_n$	pSMAD3–SMMAD4 heterodimer translocates to the nucleus
$pSMAD2_n\text{-}SMAD4_n \xrightarrow{k_{\mathrm{sma8}}} SMAD2_n\text{-}SMAD4_n$	Nuclear pSMAD2–SMAD4 is dephosphorylated
$pSMAD3_n\text{-}SMAD4_n \xrightarrow{k_{\mathrm{sma8}}} SMAD3_n\text{-}SMAD4_n$	Nuclear pSMAD3–SMAD4 is dephosphorylated
$SMAD2_n\text{-}SMAD4_n \xrightarrow{k_{\mathrm{sma9}}} SMAD2_n + SMAD4_n$	Nuclear SMAD2–SMAD4 heterodimer dissociates
$SMAD3_n\text{-}SMAD4_n \xrightarrow{k_{\mathrm{sma9}}} SMAD3_n + SMAD4_n$	Nuclear SMAD3–SMAD4 heterodimer dissociates
$SMAD2_c \underset{k_{\mathrm{sma10b}}}{\overset{k_{\mathrm{sma10a}}}{\rightleftharpoons}} SMAD2_n$	SMAD2 shuttles between cytoplasm and nucleus
$SMAD3_c \underset{k_{\mathrm{sma10b}}}{\overset{k_{\mathrm{sma10a}}}{\rightleftharpoons}} SMAD3_n$	SMAD3 shuttles between cytoplasm and nucleus
$SMAD4_c \underset{k_{\mathrm{sma11b}}}{\overset{k_{\mathrm{sma11a}}}{\rightleftharpoons}} SMAD4_n$	SMAD4 shuttles between cytoplasm and nucleus
$\emptyset \underset{k_{\mathrm{sma12b}}}{\overset{k_{\mathrm{sma12a}}}{\rightleftharpoons}} R2_s$	TGFβRII is generated and degraded
$\emptyset \underset{k_{\mathrm{sma13b}}}{\overset{k_{\mathrm{sma13a}}}{\rightleftharpoons}} ALK5_s$	ALK5 (TGFβRI) is generated and degraded
$\emptyset \underset{k_{\mathrm{sma14b}}}{\overset{k_{\mathrm{sma14a}}}{\rightleftharpoons}} SMAD2_c$	SMAD2 is generated and degraded
$\emptyset \underset{k_{\mathrm{sma14b}}}{\overset{k_{\mathrm{sma14a}}}{\rightleftharpoons}} SMAD3_c$	SMAD3 is generated and degraded
$\emptyset \underset{k_{\mathrm{sma15b}}}{\overset{k_{\mathrm{sma15a}}}{\rightleftharpoons}} SMAD4_c$	SMAD4 is generated and degraded
$ALK5_s\text{-}R2_s\text{-}TGF\beta_{\mathrm{act}} \xrightarrow{k_{\mathrm{sma16}}} \emptyset$	The surface receptor complex is degraded
$pSMAD2_c \xrightarrow{k_{\mathrm{sma17}}} pSMAD2_n$	pSMAD2 translocates to the nucleus
$pSMAD3_c \xrightarrow{k_{\mathrm{sma17}}} pSMAD3_n$	pSMAD3 translocates to the nucleus
$pSMAD2_n + SMAD4_n \underset{k_{\mathrm{sma18b}}}{\overset{k_{\mathrm{sma18a}}}{\rightleftharpoons}} pSMAD2_n\text{-}SMAD4_n$	Nuclear pSMAD2 binds and unbinds from SMAD4
$pSMAD3_n + SMAD4_n \underset{k_{\mathrm{sma18b}}}{\overset{k_{\mathrm{sma18a}}}{\rightleftharpoons}} pSMAD3_n\text{-}SMAD4_n$	Nuclear pSMAD3 binds and unbinds from SMAD4
$pSMAD2_n \xrightarrow{k_{\mathrm{sma19}}} SMAD2_n$	Nuclear pSMAD2 is dephosphorylated
$pSMAD3_n \xrightarrow{k_{\mathrm{sma19}}} SMAD3_n$	Nuclear pSMAD3 is dephosphorylated
$pSMAD2_n \xrightarrow{k_{\mathrm{sma20}}} \emptyset$	Nuclear pSMAD2 is degraded
$pSMAD3_n \xrightarrow{k_{\mathrm{sma20}}} \emptyset$	Nuclear pSMAD3 is degraded
$R2_s \underset{k_{\mathrm{sma21b}}}{\overset{k_{\mathrm{sma21a}}}{\rightleftharpoons}} R2_c$	TGFβRII moves between cytoplasm and surface
$ALK5_s \underset{k_{\mathrm{sma22b}}}{\overset{k_{\mathrm{sma22a}}}{\rightleftharpoons}} ALK5_c$	ALK5 moves between cytoplasm and surface

Table S3 (*cont.*)

Reaction	Description
$ALK5_c\text{-}R2_c\text{-}TGF\beta_{\text{act}} \xrightarrow{k_{\text{sma23}}} ALK5_s\text{-}R2_s\text{-}TGF\beta_{\text{act}}$	The receptor complex returns from the cytoplasm to the surface
$PKG_{\text{act}} + SMAD3_c \xrightarrow{k_{\text{sma24}}} PKG_{\text{act}} + pSMAD3_{c,\text{inh}}$	Active PKG inhibits SMAD3 by phosphorylating inhibitory sites that prevent SMAD4 binding
$pSMAD3_{c,\text{inh}} \xrightarrow{k_{\text{sma25}}} SMAD3_c$	Inhibited SMAD3 is restored to its active form
$pSMAD3_n\text{-}SMAD4_n \xrightarrow{k_{\text{sma26}}} pSMAD3_n\text{-}SMAD4_n + Ca$	Nuclear pSMAD3–SMAD4 heterodimer promotes calcium production via transcription of pro-calcific genes
$pSMAD2_n\text{-}SMAD4_n \xrightarrow{k_{\text{sma27}}} pSMAD2_n\text{-}SMAD4_n + Ca$	Nuclear pSMAD2–SMAD4 heterodimer promotes calcium production via transcription of pro-calcific genes

Table S4: *NO regulation model*

Reaction	Description
$\emptyset \xrightarrow{k_{\text{NO}}} NO$	NO production, with a shear-dependent rate constant
$NO + sGC \underset{k_{\text{inh1b}}}{\overset{k_{\text{inh1a}}}{\rightleftharpoons}} NO\text{-}sGC$	NO binds and unbinds from sGC
$sGC + GTP \underset{k_{\text{inh2b}}}{\overset{k_{\text{inh2a}}}{\rightleftharpoons}} sGC\text{-}GTP$	sGC binds and unbinds from GTP
$NO\text{-}sGC + GTP \underset{k_{\text{inh3b}}}{\overset{k_{\text{inh3a}}}{\rightleftharpoons}} NO\text{-}sGC\text{-}GTP$	NO–sGC complex binds and unbinds from GTP
$sGC\text{-}GTP \xrightarrow{k_{\text{inh4}}} sGC + cGMP$	sGC catalyzes conversion of GTP to cGMP
$NO\text{-}sGC\text{-}GTP \xrightarrow{k_{\text{inh5}}} NO\text{-}sGC + cGMP$	NO–sGC catalyzes conversion of GTP to cGMP
$cGMP + PDE \underset{k_{\text{inh6b}}}{\overset{k_{\text{inh6a}}}{\rightleftharpoons}} PDE\text{-}cGMP$	cGMP binds and unbinds from PDE
$PDE\text{-}cGMP \xrightarrow{k_{\text{inh7}}} PDE + GMP$	PDE hydrolyzes cGMP to GMP
$NO \xrightarrow{k_{\text{inh8}}} \emptyset$	NO degrades
$cGMP + PKG \xrightarrow{k_{\text{inh9}}} cGMP + PKG_{\text{act}}$	cGMP activates PKG
$PKG_{\text{act}} \xrightarrow{k_{\text{inh10}}} PKG$	Active PKG deactivates
$GMP \xrightarrow{k_{\text{inh11}}} GTP$	GMP recycles back to GTP

ODEs derived from the reactions in Tables S2–S4

$$[\dot{ALK5_c}] = \underbrace{k_{\text{sma22a}}[ALK5_s]}_{\text{ALK5 receptor goes into cytoplasm}} - \underbrace{k_{\text{sma22b}}[ALK5_c]}_{\text{ALK5 receptor reattaches to surface}} \tag{S1}$$

$$\begin{aligned}
[ALK5_c\text{-}\dot{R2_c}\text{-}TGF\beta_{\text{act}}] = {} & \underbrace{k_{\text{sma5}}([SMAD2_c\text{-}ALK5_c\text{-}R2_c\text{-}TGF\beta_{\text{act}}] + [SMAD3_c\text{-}ALK5_c\text{-}R2_c\text{-}TGF\beta_{\text{act}}])}_{\text{Phosphorylation of SMAD2/3}} \\
& + \underbrace{k_{\text{sma3}}[ALK5_s\text{-}R2_s\text{-}TGF\beta_{\text{act}}]}_{\text{Receptor complex detaches from cell surface into cytoplasm}} \\
& + \underbrace{k_{\text{sma4b}}([SMAD2_c\text{-}ALK5_c\text{-}R2_c\text{-}TGF\beta_{\text{act}}] + [SMAD3_c\text{-}ALK5_c\text{-}R2_c\text{-}TGF\beta_{\text{act}}])}_{\text{Receptor complex dissociates from SMAD2/3}} \\
& - \underbrace{k_{\text{sma4a}}[ALK5_c\text{-}R2_c\text{-}TGF\beta_{\text{act}}]([SMAD2_c] + [SMAD3_c])}_{\text{Receptor complex binds to SMAD2/3}} - \underbrace{k_{\text{sma23}}[ALK5_c\text{-}R2_c\text{-}TGF\beta_{\text{act}}]}_{\text{Receptor complex breaks completely}}
\end{aligned} \tag{S2}$$

$$\dot{[ALK5_s]} = \underbrace{k_{\text{sma22b}}[ALK5_c]}_{\text{ALK5 receptor reattaches to surface}} + \underbrace{k_{\text{sma13a}}}_{\text{ALK5 receptor synthesis}} + \underbrace{k_{\text{sma2b}}[ALK5_s\text{-}R2_s\text{-}TGF\beta_{\text{act}}]}_{\text{ALK5 dissociates from receptor complex}} - \underbrace{k_{\text{sma13b}}[ALK5_s]}_{\text{ALK5 receptor degradation}} - \underbrace{k_{\text{sma2a}}[ALK5_s][R2_s\text{-}TGF\beta_{\text{act}}]}_{\text{Receptor complex formation with ALK5}} - \underbrace{k_{\text{sma22a}}[ALK5_s]}_{\text{ALK5 receptor goes into cytoplasm}} \tag{S3}$$

$$\dot{[ALK5_s\text{-}R2_s\text{-}TGF\beta_{\text{act}}]} = \underbrace{k_{\text{sma2a}}[ALK5_s][R2_s\text{-}TGF\beta_{\text{act}}]}_{\text{Receptor complex formation with ALK5}} - \underbrace{k_{\text{sma2b}}[ALK5_s\text{-}R2_s\text{-}TGF\beta_{\text{act}}]}_{\text{ALK5 dissociates from receptor complex}} - \underbrace{k_{\text{sma3}}[ALK5_s\text{-}R2_s\text{-}TGF\beta_{\text{act}}]}_{\text{Receptor complex detaches from cell surface into cytoplasm}} - \underbrace{(k_{\text{sma16ld}} + k_{\text{sma16cd}})[ALK5_s\text{-}R2_s\text{-}TGF\beta_{\text{act}}]}_{\text{Ligand induced and constitutive degradation of receptor complex}} \tag{S4}$$

$$\dot{[Ca]} = \underbrace{k_{\text{sma26}}[pSMAD3_n\text{-}SMAD4_n]}_{\text{pSMAD3–4 transcription of calcific genes}} + \underbrace{k_{\text{sma27}}[pSMAD2_n\text{-}SMAD4_n]}_{\text{pSMAD2–4 transcription of calcific genes}} \tag{S5}$$

$$\dot{[FoamCell]} = \underbrace{k_{\text{inf4}}[oxLDL][Macrophage]}_{\text{Foam cell formation}} - \underbrace{k_{\text{inf9}}[FoamCell]}_{\text{Foam cell apoptosis}} \tag{S6}$$

$$\dot{[sGC]} = \underbrace{(k_{\text{inh4}} + k_{\text{inh2b}})[sGC\text{-}GTP]}_{\text{GTP unbinds or cGMP made}} + \underbrace{k_{\text{inh1b}}[NO\text{-}sGC]}_{\text{NO unbinds from sGC}} - \underbrace{k_{\text{inh2a}}[sGC][GTP]}_{\text{GTP binds sGC}} - \underbrace{k_{\text{inh1a}}[NO][sGC]}_{\text{NO binds sGC}} \tag{S7}$$

$$\dot{[sGC\text{-}GTP]} = \underbrace{k_{\text{inh2a}}[sGC][GTP]}_{\text{GTP binds sGC}} - \underbrace{(k_{\text{inh4}} + k_{\text{inh2b}})[sGC\text{-}GTP]}_{\text{cGMP made or GTP unbinds}} \tag{S8}$$

$$\dot{[GMP]} = \underbrace{k_{\text{inh7}}[PDE\text{-}cGMP]}_{\text{GMP made from cGMP–PDE complex}} - \underbrace{k_{\text{inh11}}[GMP]}_{\text{GMP recycled back to GTP}} \tag{S9}$$

$$\dot{[cGMP]} = \underbrace{k_{\text{inh5}}[NO\text{-}sGC\text{-}GTP]}_{\text{cGMP made from NO–sGC–GTP}} + \underbrace{k_{\text{inh4}}[sGC\text{-}GTP]}_{\text{cGMP made from sGC–GTP}} + \underbrace{k_{\text{inh6b}}[PDE\text{-}cGMP]}_{\text{cGMP unbinds from PDE}} - \underbrace{k_{\text{inh6a}}[cGMP][PDE]}_{\text{cGMP binds PDE}} \tag{S10}$$

$$\dot{[GTP]} = \underbrace{k_{\text{inh3b}}[NO\text{-}sGC\text{-}GTP]}_{\text{GTP unbinds from NO–sGC}} + \underbrace{k_{\text{inh2b}}[sGC\text{-}GTP]}_{\text{GTP unbinds from sGC}} + \underbrace{k_{\text{kinh11}}[GMP]}_{\text{GMP recycled back to GTP}} - \underbrace{k_{\text{inh2a}}[sGC][GTP]}_{\text{GTP binds sGC}} - \underbrace{k_{\text{inh3a}}[GTP][NO\text{-}sGC]}_{\text{GTP binds NO–sGC}} \tag{S11}$$

$$\dot{[LDL]} = \underbrace{k_{\text{inf1}}(\bar{\tau})}_{\text{Subendothelial LDL penetration}} - \underbrace{k_{\text{inf2}}[LDL]}_{\text{LDL diffusing out}} - \underbrace{k_{\text{inf3}}[LDL]}_{\text{LDL oxidation}} \tag{S12}$$

$$\dot{[oxLDL]} = \underbrace{k_{\text{inf3}}[LDL]}_{\text{LDL oxidation}} - \underbrace{k_{\text{inf4}}[oxLDL][Macrophage]}_{\text{Foam cell formation}} \tag{S13}$$

$$\dot{[Macrophage]} = \underbrace{k_{\text{inf6}}[Monocyte]}_{\text{Monocyte differentiation to macrophage}} - \underbrace{k_{\text{inf4}}[oxLDL][Macrophage]}_{\text{Foam cell formation}} - \underbrace{k_{\text{inf13}}[Macrophage]}_{\text{Macrophage apoptosis}} \tag{S14}$$

$$\dot{[Monocyte]} = \underbrace{k_{\text{inf5}}(\bar{\tau})[oxLDL]}_{\text{Baseline monocyte capture}} - \underbrace{k_{\text{inf6}}[Monocyte]}_{\text{Monocyte differentiation to macrophage}} - \underbrace{k_{\text{inf7}}[Monocyte]}_{\text{Monocyte apoptosis}} \tag{S15}$$

$$\dot{[NO]} = \underbrace{k_{\text{NO}}(\tau)}_{\text{Flow-induced NO production}} + \underbrace{k_{\text{inh1b}}[NO\text{-}sGC]}_{\text{NO unbinds from sGC}} - \underbrace{k_{\text{inh8}}[NO]}_{\text{NO degraded}} - \underbrace{k_{\text{inh1a}}[NO][sGC]}_{\text{NO binds to sGC}} \tag{S16}$$

$$\dot{[NO\text{-}sGC]} = \underbrace{k_{\text{inh1a}}[NO][sGC]}_{\text{NO binds sGC}} + \underbrace{(k_{\text{inh3b}} + k_{\text{inh5}})[NO\text{-}sGC\text{-}GTP]}_{\text{GTP unbinds or cGMP made}} - \underbrace{k_{\text{inh1b}}[NO\text{-}sGC]}_{\text{NO unbinds from sGC}} - \underbrace{k_{\text{inh3a}}[GTP][NO\text{-}sGC]}_{\text{GTP binds NO–sGC complex}} \tag{S17}$$

$$\dot{[NO\text{-}sGC\text{-}GTP]} = \underbrace{k_{\text{inh3a}}[GTP][NO\text{-}sGC]}_{\text{GTP binds NO–sGC complex}} - \underbrace{(k_{\text{inh3b}} + k_{\text{inh5}})[NO\text{-}sGC\text{-}GTP]}_{\text{GTP unbinds or cGMP made}} \tag{S18}$$

$$\dot{[PDE]} = \underbrace{(k_{\text{inh7}} + k_{\text{inh6b}})[PDE\text{-}cGMP]}_{\text{PDE released from cGMP–PDE}} - \underbrace{k_{\text{inh6a}}[cGMP][PDE]}_{\text{cGMP binds PDE}} \tag{S19}$$

$$\dot{[PDE\text{-}cGMP]} = \underbrace{k_{\text{inh6a}}[cGMP][PDE]}_{\text{cGMP binds PDE}} - \underbrace{(k_{\text{inh7}} + k_{\text{inh6b}})[PDE\text{-}cGMP]}_{\text{GMP made or cGMP unbinds}} \tag{S20}$$

$$\dot{[PKG]} = \underbrace{k_{\text{inh10}}[PKG_{\text{act}}]}_{\text{PKG autoinhibitory deactivation}} - \underbrace{k_{\text{inh9}}[PKG][cGMP]}_{\text{cGMP activates PKG}} \tag{S21}$$

$$\dot{[PKG_{\text{act}}]} = \underbrace{k_{\text{inh9}}[PKG][cGMP]}_{\text{cGMP activates PKG}} - \underbrace{k_{\text{inh10}}[PKG_{\text{act}}]}_{\text{PKG deactivates}} \tag{S22}$$

$$\dot{[R2_c]} = \underbrace{k_{\text{sma21a}}[R2_s]}_{\text{R2 receptor goes into cytoplasm}} - \underbrace{k_{\text{sma21b}}[R2_c]}_{\text{R2 receptor reattaches to surface}} \tag{S23}$$

$$\begin{aligned}\dot{[R2_s]} = {} & \underbrace{k_{\text{sma12a}}}_{\text{R2 receptor synthesis}} + \underbrace{k_{\text{sma23}}[ALK5_c\text{-}R2_c\text{-}TGF\beta_{\text{act}}]}_{\text{Receptor complex breaks completely}} + \underbrace{k_{\text{sma21b}}[R2_c]}_{\text{R2 receptor reattaches to surface}} \\ & + \underbrace{k_{\text{sma1b}}[R2_s\text{-}TGF\beta_{\text{act}}]}_{\text{TGF-}\beta\text{ unbinds from R2 receptor}} - \underbrace{k_{\text{sma12b}}[R2_s]}_{\text{R2 receptor degradation}} - \underbrace{k_{\text{sma1a}}[R2_s][TGF\beta_{\text{act}}]}_{\text{TGF-}\beta\text{ binds to R2 receptor}} - \underbrace{k_{\text{sma21a}}[R2_s]}_{\text{R2 receptor goes into cytoplasm}}\end{aligned} \tag{S24}$$

$$\begin{aligned}\dot{[R2_s\text{-}TGF\beta_{\text{act}}]} = {} & \underbrace{k_{\text{sma2b}}[ALK5_s\text{-}R2_s\text{-}TGF\beta_{\text{act}}]}_{\text{ALK5 dissociates from receptor complex}} + \underbrace{k_{\text{sma1a}}[R2_s][TGF\beta_{\text{act}}]}_{\text{TGF-}\beta\text{ binds to R2 receptor}} \\ & - \underbrace{k_{\text{sma2a}}[ALK5_s][R2_s\text{-}TGF\beta_{\text{act}}]}_{\text{Receptor complex formation with ALK5}} - \underbrace{k_{\text{sma1b}}[R2_s\text{-}TGF\beta_{\text{act}}]}_{\text{TGF-}\beta\text{ unbinds from R2 receptor}}\end{aligned} \tag{S25}$$

$$\begin{aligned}\dot{[SMAD2_c]} = {} & \underbrace{k_{\text{sma14a}}}_{\text{SMAD2/3 synthesis}} + \underbrace{k_{\text{sma4b}}[SMAD2_c\text{-}ALK5_c\text{-}R2_c\text{-}TGF\beta_{\text{act}}]}_{\text{Receptor complex dissociates from SMAD2/3}} + \underbrace{k_{\text{sma10b}}[SMAD2_n]}_{\text{Translocation out of nucleus}} \\ & - \underbrace{k_{\text{sma14b}}[SMAD2_c]}_{\text{SMAD2/3 degradation}} - \underbrace{k_{\text{sma4a}}[SMAD2_c][ALK5_c\text{-}R2_c\text{-}TGF\beta_{\text{act}}]}_{\text{Receptor complex binds to SMAD2/3}} - \underbrace{k_{\text{sma10a}}[SMAD2_c]}_{\text{Translocation into nucleus}}\end{aligned} \tag{S26}$$

$$\dot{[pSMAD2_c]} = \underbrace{k_{\text{sma5}}[SMAD2_c\text{-}ALK5_c\text{-}R2_c\text{-}TGF\beta_{\text{act}}]}_{\text{Receptor complex phosphorylates SMAD2/3}} + \underbrace{k_{\text{sma6b}}[pSMAD2_c\text{-}SMAD4_c]}_{\text{pSMAD2/3 unbinds from SMAD4}} - \underbrace{k_{\text{sma6a}}[SMAD4_c][pSMAD2_c]}_{\text{pSMAD2/3 binds to SMAD4 to form pSMAD2/3-4 complex}} - \underbrace{k_{\text{sma17}}[pSMAD2_c]}_{\text{pSMAD2/3 translocates into nucleus}} \tag{S27}$$

$$\dot{[SMAD2_c\text{-}ALK5_c\text{-}R2_c\text{-}TGF\beta_{\text{act}}]} = \underbrace{k_{\text{sma4a}}[SMAD2_c][ALK5_c\text{-}R2_c\text{-}TGF\beta_{\text{act}}]}_{\text{Receptor complex binds to SMAD2/3}} - \underbrace{(k_{\text{sma4b}} + k_{\text{sma5}})[SMAD2_c\text{-}ALK5_c\text{-}R2_c\text{-}TGF\beta_{\text{act}}]}_{\text{Dissociation and phosphorylation of SMAD2/3}} \tag{S28}$$

$$\dot{[pSMAD2_c\text{-}SMAD4_c]} = \underbrace{k_{\text{sma6a}}[SMAD4_c][pSMAD2_c]}_{\text{pSMAD2/3 binds to SMAD4}} - \underbrace{k_{\text{sma6b}}[pSMAD2_c\text{-}SMAD4_c]}_{\text{pSMAD2/3 unbinds from SMAD4}} - \underbrace{k_{\text{sma7}}[pSMAD2_c\text{-}SMAD4_c]}_{\text{pSMAD2/3-4 complex translocates into nucleus}} \tag{S29}$$

$$\dot{[SMAD2_n]} = \underbrace{k_{\text{sma19}}[pSMAD2_n]}_{\text{Dephosphorylation}} + \underbrace{k_{\text{sma9}}[SMAD2_n\text{-}SMAD4_n]}_{\text{Complex breaks}} + \underbrace{k_{\text{sma10a}}[SMAD2_c]}_{\text{Translocation into nucleus}} - \underbrace{k_{\text{sma10b}}[SMAD2_n]}_{\text{Translocation out of nucleus}} \tag{S30}$$

$$\dot{[pSMAD2_n]} = \underbrace{k_{\text{sma17}}[pSMAD2_c]}_{\text{Translocation into nucleus}} + \underbrace{k_{\text{sma18b}}[pSMAD2_n\text{-}SMAD4_n]}_{\text{Complex breaks}} - \underbrace{k_{\text{sma18a}}[pSMAD2_n][SMAD4_n]}_{\text{Binding to SMAD4}} - \underbrace{k_{\text{sma19}}[pSMAD2_n]}_{\text{Dephosphorylation}} - \underbrace{k_{\text{sma20}}[pSMAD2_n]}_{\text{Degradation}} \tag{S31}$$

$$\dot{[SMAD2_n\text{-}SMAD4_n]} = \underbrace{k_{\text{sma8}}[pSMAD2_n\text{-}SMAD4_n]}_{\text{Dephosphorylation}} - \underbrace{k_{\text{sma9}}[SMAD2_n\text{-}SMAD4_n]}_{\text{SMAD2/3-4 complex breaks}} \tag{S32}$$

$$\dot{[pSMAD2_n\text{-}SMAD4_n]} = \underbrace{k_{\text{sma18a}}[pSMAD2_n][SMAD4_n]}_{\text{Nuclear pSMAD2/3 binds to SMAD4}} + \underbrace{k_{\text{sma7}}[pSMAD2_c\text{-}SMAD4_c]}_{\text{Translocation into nucleus}} - \underbrace{k_{\text{sma8}}[pSMAD2_n\text{-}SMAD4_n]}_{\text{Dephosphorylation of pSMAD2/3-4 complex}} - \underbrace{k_{\text{sma18b}}[pSMAD2_n\text{-}SMAD4_n]}_{\text{Nuclear complex breaks}} \tag{S33}$$

$$\dot{[SMAD3_c]} = \underbrace{k_{\text{sma14a}}}_{\text{SMAD2/3 synthesis}} + \underbrace{k_{\text{sma4b}}[SMAD3_c\text{-}ALK5_c\text{-}R2_c\text{-}TGF\beta_{\text{act}}]}_{\text{Receptor complex dissociates from SMAD2/3}} + \underbrace{k_{\text{sma10b}}[SMAD3_n]}_{\text{Translocation out of nucleus}} + \underbrace{k_{\text{sma25}}[pSMAD3_{c,\text{inh}}]}_{\text{Cytosolic pSMAD3 desphosphorylates}} - \underbrace{k_{\text{sma14b}}[SMAD3_c]}_{\text{SMAD2/3 degradation}} - \underbrace{k_{\text{sma4a}}[SMAD3_c][ALK5_c\text{-}R2_c\text{-}TGF\beta_{\text{act}}]}_{\text{Receptor complex binds to SMAD2/3}} - \underbrace{k_{\text{sma24}}[PKG_{\text{act}}][SMAD3_c]}_{\text{PKG inhibits SMAD3 via hyperphosphorylation}} - \underbrace{k_{\text{sma10a}}[SMAD3_c]}_{\text{Translocation into nucleus}} \tag{S34}$$

$$\dot{[pSMAD3_c]} = \underbrace{k_{\text{sma5}}[SMAD3_c\text{-}ALK5_c\text{-}R2_c\text{-}TGF\beta_{\text{act}}]}_{\text{Receptor complex phosphorylates SMAD2/3}} + \underbrace{k_{\text{sma6b}}[pSMAD3_c\text{-}SMAD4_c]}_{\text{pSMAD2/3 unbinds from SMAD4}} - \underbrace{k_{\text{sma6a}}[SMAD4_c][pSMAD3_c]}_{\text{pSMAD2/3 binds to SMAD4 to form pSMAD2/3-4 complex}} - \underbrace{k_{\text{sma17}}[pSMAD3_c]}_{\text{pSMAD2/3 translocates into nucleus}} \tag{S35}$$

$$\dot{[pSMAD3_{c,\mathrm{inh}}]} = \underbrace{k_{\mathrm{sma24}}[PKG_{\mathrm{act}}][SMAD3_c]}_{\text{PKG inhibits SMAD3 via hyperphosphorylation}} - \underbrace{k_{\mathrm{sma25}}[pSMAD3_{c,\mathrm{inh}}]}_{\text{Cytosolic inhibited pSMAD3 desphosphorylates}} \tag{S36}$$

$$\begin{aligned}\dot{[SMAD3_c\text{-}ALK5_c\text{-}R2_c\text{-}TGF\beta_{\mathrm{act}}]} = {} & \underbrace{k_{\mathrm{sma4a}}[SMAD3_c][ALK5_c\text{-}R2_c\text{-}TGF\beta_{\mathrm{act}}]}_{\text{Receptor complex binds to SMAD2/3}} \\ & - \underbrace{(k_{\mathrm{sma4b}} + k_{\mathrm{sma5}})[SMAD3_c\text{-}ALK5_c\text{-}R2_c\text{-}TGF\beta_{\mathrm{act}}]}_{\text{Dissociation and phosphorylation of SMAD2/3}}\end{aligned} \tag{S37}$$

$$\begin{aligned}\dot{[pSMAD3_c\text{-}SMAD4_c]} = {} & \underbrace{k_{\mathrm{sma6a}}[SMAD4_c][pSMAD3_c]}_{\text{pSMAD2/3 binds to SMAD4}} - \underbrace{k_{\mathrm{sma6b}}[pSMAD2_c\text{-}SMAD4_c]}_{\text{pSMAD2/3 unbinds from SMAD4}} \\ & - \underbrace{k_{\mathrm{sma7}}[pSMAD3_c\text{-}SMAD4_c]}_{\text{pSMAD2/3-4 complex translocates into nucleus}}\end{aligned} \tag{S38}$$

$$\dot{[SMAD3_n]} = \underbrace{k_{\mathrm{sma19}}[pSMAD3_n]}_{\text{Dephosphorylation}} + \underbrace{k_{\mathrm{sma9}}[SMAD3_n\text{-}SMAD4_n]}_{\text{Complex breaks}} + \underbrace{k_{\mathrm{sma10a}}[SMAD3_c]}_{\text{Translocation into nucleus}} - \underbrace{k_{\mathrm{sma10b}}[SMAD3_n]}_{\text{Translocation out of nucleus}} \tag{S39}$$

$$\begin{aligned}\dot{[pSMAD3_n]} = {} & \underbrace{k_{\mathrm{sma17}}[pSMAD3_c]}_{\text{Translocation into nucleus}} + \underbrace{k_{\mathrm{sma18b}}[pSMAD3_n\text{-}SMAD4_n]}_{\text{Complex breaks}} - \underbrace{k_{\mathrm{sma18a}}[pSMAD3_n][SMAD4_n]}_{\text{Binding to SMAD4}} \\ & - \underbrace{k_{\mathrm{sma19}}[pSMAD3_n]}_{\text{Dephosphorylation}} - \underbrace{k_{\mathrm{sma20}}[pSMAD3_n]}_{\text{Degradation}}\end{aligned} \tag{S40}$$

$$\dot{[SMAD3_n\text{-}SMAD4_n]} = \underbrace{k_{\mathrm{sma8}}[pSMAD3_n\text{-}SMAD4_n]}_{\text{Dephosphorylation}} - \underbrace{k_{\mathrm{sma9}}[SMAD3_n\text{-}SMAD4_n]}_{\text{SMAD2/3-4 complex breaks}} \tag{S41}$$

$$\begin{aligned}\dot{[pSMAD3_n\text{-}SMAD4_n]} = {} & \underbrace{k_{\mathrm{sma18a}}[pSMAD3_n][SMAD4_n]}_{\text{Nuclear pSMAD2/3 binds to SMAD4}} + \underbrace{k_{\mathrm{sma7}}[pSMAD3_c\text{-}SMAD4_c]}_{\text{Translocation into nucleus}} \\ & - \underbrace{k_{\mathrm{sma8}}[pSMAD3_n\text{-}SMAD4_n]}_{\text{Dephosphorylation of pSMAD2/3-4 complex}} - \underbrace{k_{\mathrm{sma18b}}[pSMAD3_n\text{-}SMAD4_n]}_{\text{Nuclear complex breaks}}\end{aligned} \tag{S42}$$

$$\begin{aligned}\dot{[SMAD4_c]} = {} & \underbrace{k_{\mathrm{sma15a}}}_{\text{SMAD4 synthesis}} + \underbrace{k_{\mathrm{sma6b}}([pSMAD2_c\text{-}SMAD4_c] + [pSMAD3_c\text{-}SMAD4_c])}_{\text{pSMAD2/3 unbinds from SMAD4}} \\ & + \underbrace{k_{\mathrm{sma11b}}[SMAD4_n]}_{\text{SMAD4 translocates out of nucleus}} - \underbrace{k_{\mathrm{sma15b}}[SMAD4_c]}_{\text{SMAD4 degradation}} - \underbrace{k_{\mathrm{sma6a}}[SMAD4_c]([pSMAD2_c] + [pSMAD3_c])}_{\text{pSMAD2/3 binds to SMAD4 to form pSMAD2/3-4 complex}} \\ & - \underbrace{k_{\mathrm{sma11a}}[SMAD4_c]}_{\text{SMAD4 translocates into nucleus}}\end{aligned} \tag{S43}$$

$$\begin{aligned}\dot{[SMAD4_n]} = {} & \underbrace{k_{\mathrm{sma9}}([SMAD2_n\text{-}SMAD4_n] + [SMAD3_n\text{-}SMAD4_n])}_{\text{Complex breaks}} \\ & + \underbrace{k_{\mathrm{sma18b}}([pSMAD2_n\text{-}SMAD4_n] + [pSMAD3_n\text{-}SMAD4_n])}_{\text{Nuclear complex breaks}} + \underbrace{k_{\mathrm{sma11a}}[SMAD4_c]}_{\text{SMAD4 translocates into nucleus}} \\ & - \underbrace{k_{\mathrm{sma11b}}[SMAD4_n]}_{\text{SMAD4 translocates out of nucleus}} - \underbrace{k_{\mathrm{sma18a}}([pSMAD2_n] + [pSMAD3_n])[SMAD4_n]}_{\text{Nuclear pSMAD2/3 binds to SMAD4}}\end{aligned} \tag{S44}$$

$$[\dot{TGF\beta}] = \underbrace{k_{\text{inf10}}}_{\text{LatentTGF-}\beta\text{ production}} - \underbrace{k_{\text{inf12}}[TGF\beta]}_{\text{LatentTGF-}\beta\text{ degradation}} - \underbrace{k_{\text{inf8}}([FoamCell] + [Macrophage])[TGF\beta]}_{\text{TGF-}\beta\text{ activation}} \tag{S45}$$

$$[\dot{TGF\beta}_{\text{act}}] = \underbrace{k_{\text{inf8}}([FoamCell] + [Macrophage])[TGF\beta]}_{\text{TGF-}\beta\text{ activation}} + \underbrace{k_{\text{sma1b}}[R2_s\text{-}TGF\beta_{\text{act}}]}_{\text{TGF-}\beta\text{ unbinds from R2 receptor}}$$
$$+ \underbrace{k_{\text{sma23}}[ALK5_c\text{-}R2_c\text{-}TGF\beta_{\text{act}}]}_{\text{Receptor complex breaks completely}} - \underbrace{k_{\text{sma1a}}[R2_s][TGF\beta_{\text{act}}]}_{\text{TGF-}\beta\text{ binds to R2 receptor}} - \underbrace{k_{\text{inf11}}[TGF\beta_{\text{act}}]}_{\text{ActiveTGF-}\beta\text{ degradation}} \tag{S46}$$

Initial species concentrations and rate constants

Table S5: ***Initial species concentrations for the integrated SB model***

Species	Initial Amount (M)	Module(s)	Reference
$ALK5_c$	1.19×10^{-11}	SMAD signaling	Chung et al.[1]
$ALK5_s$	1.33×10^{-12}	SMAD signaling	Chung et al.[1]
$FoamCell$	0	Inflammation	Arzani et al.[2]
sGC	3.2×10^{-10}	NO regulation	Garmaroudi et al.[3]
$cGMP$	0	NO regulation	Garmaroudi et al.[3]
GTP	9.75×10^{-7}	NO regulation	Garmaroudi et al.[3]
LDL	0	Inflammation	Arzani et al.[2]
$oxLDL$	0	Inflammation	Arzani et al.[2]
$Macrophage$	0	Inflammation	Arzani et al.[2]
$Monocyte$	0	Inflammation	Arzani et al.[2]
NO	3.84×10^{-11}	NO regulation	He and Liu[4]
PDE	4.8×10^{-10}	NO regulation	Garmaroudi et al.[3]
PKG	3.2×10^{-8}	NO regulation	Inferred from Keilbach et al.[5]
PKG_{act}	0	SMAD signaling, NO regulation	N/A
$R2_c$	1.19×10^{-11}	SMAD signaling	Chung et al.[1]
$R2_s$	1.33×10^{-12}	SMAD signaling	Chung et al.[1]
$SMAD2_c$	2.26×10^{-10}	SMAD signaling	Chung et al.[1]
$SMAD2_n$	3.98×10^{-11}	SMAD signaling	Chung et al.[1]
$SMAD3_c$	2.26×10^{-10}	SMAD signaling	Chung et al.[1]
$SMAD3_n$	3.98×10^{-11}	SMAD signaling	Chung et al.[1]
$SMAD4_c$	3.31×10^{-10}	SMAD signaling	Chung et al.[1]
$SMAD4_n$	3.45×10^{-11}	SMAD signaling	Chung et al.[1]
$TGF\beta$	0	Inflammation	Arzani et al.[2]
$TGF\beta_{\text{act}}$	0	Inflammation, SMAD signaling	Arzani et al.[2]

In Table S5, the initial concentrations of PKG and PKG_{act} are based on estimates from Keilbach et al.,[5] who reported tissue concentrations for PKG of ~10 pmol/g of wet tissue. Assuming a tissue density of 1 g/mL, this corresponds to a tissue concentration on the order of 10^{-8} M. Because the reported values in the study were somewhat higher, we adopt this estimate as the total PKG concentration and set the initial concentration of PKG accordingly. The active form, PKG_{act}, is initialized at 0.

Table S6: *Rate constants for the inflammation pathway model*

Parameter	Value	Definition	Reference
$\overline{k}_{\text{inf1}}$	3.78×10^{-6} M/s	Baseline LDL penetration	Arzani et al.[2]
k_{inf1}	*See Eq.* (S47)	Subendothelial LDL penetration	Arzani et al.[2]
k_{inf2}	2.40×10^{-5} s^{-1}	LDL out diffusion	Arzani et al.[2]
k_{inf3}	3×10^{-4} s^{-1}	LDL oxidation	Arzani et al.[2]
k_{inf4}	1.71×10^{-16} M^{-1}s^{-1}	Foam cell formation	Arzani et al.[2]
$\overline{k}_{\text{inf5}}$	6.37×10^{5} s^{-1}	Baseline monocyte capture	Arzani et al.[2]
k_{inf5}	*See Eq.* (S48)	Monocyte capture via LDL	Arzani et al.[2]
k_{inf6}	1.15×10^{-6} s^{-1}	Monocyte $\rightarrow$ macrophage differentiation	Arzani et al.[2]
k_{inf7}	2.76×10^{-6} s^{-1}	Monocyte apoptosis	Arzani et al.[2]
k_{inf8}	7.41×10^{-13} M^{-1}s^{-1}	TGF-β activation	Arzani et al.[2]
k_{inf9}	1.22×10^{-8} s^{-1}	Foam cell apoptosis	Inferred from Jiang et al.[6] and Bajpai et al.[7]
k_{inf10}	*See Eq.* (S49)	Latent TGF-β production	Arzani et al.[2]
k_{inf11}	5.78×10^{-3} s^{-1}	Active TGF-β degradation	Arzani et al.[2]
k_{inf12}	1.28×10^{-4} s^{-1}	Latent TGF-β degradation	Arzani et al.[2]
k_{inf13}	1.32×10^{-9} s^{-1}	Macrophage apoptosis	Estimated from Bajpai et al.[7]

In Table S6, k_{inf1} is based on Arzani et al.,[2] who reported a baseline LDL penetration rate, which we denote as $\overline{k}_{\text{inf1}}$. This value is multiplied by a factor $(1+\bar{\tau}/\bar{\tau}_{\text{ref}})^{-1}$ to account for the effects of WSS on LDL penetration, i.e.,

$$k_{\text{inf1}}(\tau) = \frac{\overline{k}_{\text{inf1}}}{1+\bar{\tau}/\bar{\tau}_{\text{ref}}}. \tag{S47}$$

Similarly, k_{inf5} is based on the second-order rate constant reported by Arzani et al.[2] for the interaction between monocytes and LDL. We multiply this by the same scaling factor to account for variations in WSS, i.e.,

$$k_{\text{inf5}}(\tau) = \frac{\overline{k}_{\text{inf5}}}{1+\bar{\tau}/\bar{\tau}_{\text{ref}}}. \tag{S48}$$

Additionally, we define the rate constant for foam cell apoptosis as $k_{\text{inf9}} = 9.28 \cdot k_{\text{inf13}}$, where k_{inf13} is the macrophage apoptosis rate constant. This is based on results from Bajpai et al.,[7] who estimated the percentage of sex-mismatched CCR2^{+} macrophages in the body after 8.8 years. They also reported that oxLDL-treated macrophages had a 47.7% apoptosis rate after 48h, compared to 5.14% in control, which is a ∼9.28-fold increase in the apoptosis rate for foam cells.

Finally, the rate constant for latent TGF-β production, k_{inf10}, is assumed to depend on the total concentration of immune cells (*FoamCell* + *Macrophage*) and V_{valve} (Table 1), which assumes different values depending on valve thickness,

$$k_{\text{inf10}} = \alpha \cdot \frac{3.3 \times 10^{-7} \cdot ([\mathit{FoamCell}] + [\mathit{Macrophage}]) \cdot V_{\text{valve}}}{\alpha \cdot ([\mathit{FoamCell}] + [\mathit{Macrophage}]) \cdot V_{\text{valve}} \cdot \psi + 2.84 \times 10^{4}}. \tag{S49}$$

Here, α is the foam cell formation ratio (See Table 1) and ψ is a unit conversion factor defined as *Desired units* = *Current units*/ψ. In Arzani et al.,[2] immune cell concentrations are in g/m^3 and V_{valve} is in m^3. This gives k_{inf10} in units of g/(s·m^3). The conversion factor ψ is used here to convert k_{inf10} into units of M/s, consistent with the other sub-models in the SB model.

Table S7: ***Rate constants for the SMAD signaling pathway model***

Parameter	Value	Description	Reference
k_{sma1a}	4.14×10^{10} $M^{-1}s^{-1}$	TGF-β binds to R2 receptor	Chung et al.[1]
k_{sma1b}	4.97×10^{-3} s^{-1}	TGF-β unbinds from R2 receptor	Chung et al.[1]
k_{sma2a}	4.14×10^{10} $M^{-1}s^{-1}$	Receptor complex formation with ALK5	Chung et al.[1]
k_{sma2b}	4.97×10^{-3} s^{-1}	ALK5 dissociates from receptor complex	Chung et al.[1]
k_{sma3}	6.58×10^{-3} s^{-1}	Receptor complex detaches from the cell surface	Chung et al.[1]
k_{sma4a}	9.42×10^{8} $M^{-1}s^{-1}$	Receptor complex binds to SMAD2/3	Chung et al.[1]
k_{sma4b}	1.62×10^{-2} s^{-1}	Receptor complex dissociates from SMAD2/3	Chung et al.[1]
k_{sma5}	7.47×10^{2} s^{-1}	Receptor complex phosphorylates SMAD2/3	Chung et al.[1]
k_{sma6a}	3.77×10^{10} $M^{-1}s^{-1}$	pSMAD2/3 binds to SMAD4 to form pSMAD2/3-4	Chung et al.[1]
k_{sma6b}	2.43×10^{1} s^{-1}	pSMAD2/3 unbinds from SMAD4	Chung et al.[1]
k_{sma7}	1.35×10^{-2} s^{-1}	pSMAD2/3-4 complex translocates into nucleus	Chung et al.[1]
k_{sma8}	4.2×10^{-4} s^{-1}	pSMAD2/3-4 complex dephosphorylates	Chung et al.[1]
k_{sma9}	1.68×10^{-3} s^{-1}	SMAD2/3-4 complex breaks	Chung et al.[1]
k_{sma10a}	2.7×10^{-3} s^{-1}	SMAD2/3 translocates into nucleus	Chung et al.[1]
k_{sma10b}	5.8×10^{-3} s^{-1}	SMAD2/3 translocates out of nucleus	Chung et al.[1]
k_{sma11a}	3.35×10^{-4} s^{-1}	SMAD4 translocates into nucleus	Chung et al.[1]
k_{sma11b}	2.9×10^{-3} s^{-1}	SMAD4 translocates out of nucleus	Chung et al.[1]
k_{sma12a}	3.54×10^{-16} M/s	R2 receptor synthesis	Chung et al.[1]
k_{sma12b}	4.67×10^{-4} s^{-1}	R2 receptor degradation	Chung et al.[1]
k_{sma13a}	3.54×10^{-16} M/s	ALK5 receptor synthesis	Chung et al.[1]
k_{sma13b}	4.67×10^{-4} s^{-1}	ALK5 receptor degradation	Chung et al.[1]
k_{sma14a}	1.21×10^{-15} M/s	SMAD2/3 synthesis	Chung et al.[1]
k_{sma14b}	1.08×10^{-5} s^{-1}	SMAD2/3 degradation	Chung et al.[1]
k_{sma15a}	2.21×10^{-15} M/s	SMAD4 synthesis	Chung et al.[1]
k_{sma15b}	2×10^{-5} s^{-1}	SMAD4 degradation	Chung et al.[1]
$k_{sma16ld}$	6.58×10^{-3} s^{-1}	Ligand induced degradation of receptor complex	Chung et al.[1]
$k_{sma16cd}$	4.67×10^{-4} s^{-1}	Constitutive degradation of receptor complex	Chung et al.[1]
k_{sma17}	8.38×10^{-3} s^{-1}	pSMAD2/3 translocates into nucleus	Chung et al.[1]
k_{sma18a}	1.05×10^{9} $M^{-1}s^{-1}$	Nuclear pSMAD2/3 binds to SMAD4	Chung et al.[1]
k_{sma18b}	1.52×10^{-2} s^{-1}	Nuclear pSMAD2/3-4 complex breaks	Chung et al.[1]
k_{sma19}	4.2×10^{-4} s^{-1}	Nuclear pSMAD2/3 dephosphorylates	Chung et al.[1]
k_{sma20}	9×10^{-5} s^{-1}	Nuclear SMAD2/3 degradation	Chung et al.[1]
k_{sma21a}	6.58×10^{-3} s^{-1}	R2 receptor goes into cytoplasm	Chung et al.[1]
k_{sma21b}	6.58×10^{-4} s^{-1}	R2 receptor reattaches to surface	Chung et al.[1]
k_{sma22a}	6.58×10^{-3} s^{-1}	ALK5 receptor goes into cytoplasm	Chung et al.[1]
k_{sma22b}	6.58×10^{-4} s^{-1}	ALK5 receptor reattaches to surface	Chung et al.[1]
k_{sma23}	6.58×10^{-4} s^{-1}	Receptor complex breaks completely	Chung et al.[1]
k_{sma24}	5×10^{3} $M^{-1}s^{-1}$	PKG inhibits SMAD3 via hyperphosphorylation	Chung et al.[1]
k_{sma25}	4.20×10^{-4} s^{-1}	Cytosolic inhibited pSMAD3 desphosphorylates	Chung et al.[1]
k_{sma26}	*See Eq.* (**??**)	pSMAD3-4 transcribes pro-calcific genes	N/A
k_{sma27}	*See Eq.* (**??**)	pSMAD2-4 transcribes pro-calcific genes	N/A

Table S8: ***Rate constants for the NO regulation model***

Parameter	Value	Description	Reference
k_{NO}	*See Fig. S1*	NO synthesis	Sriram et al.[8]
k_{inh1a}	0.78×10^6 M^{-1}s^{-1}	NO binds to sGC	Inferred from Sharina and Martin[9]
k_{inh1b}	1.8×10^{-1} s^{-1}	NO unbinds from sGC	Sayed et al.[10]
k_{inh2a}	2.5×10^5 M^{-1}s^{-1}	GTP binds to sGC	*See text below*
k_{inh2b}	1.8×10^2 s^{-1}	GTP unbinds from sGC	Garmaroudi et al.[3]
k_{inh3a}	3.13×10^7 M^{-1}s^{-1}	GTP binds to NO-sGC	*See text below*
k_{inh3b}	4.3×10 s^{-1}	GTP unbinds from NO-sGC	Garmaroudi et al.[3]
k_{inh4}	1.5×10^{-1} s^{-1}	cGMP made from GTP-sGC	Garmaroudi et al.[3]
k_{inh5}	2.87×10 s^{-1}	cGMP made from NO-GTP-sGC	Garmaroudi et al.[3]
k_{inh6a}	3.13×10^8 M^{-1}s^{-1}	cGMP binds to PDE	Garmaroudi et al.[3]
k_{inh6b}	1.30×10^{-1} s^{-1}	cGMP unbinds from PDE	Garmaroudi et al.[3]
k_{inh7}	2.2 s^{-1}	GMP made from cGMP-PDE	Garmaroudi et al.[3]
k_{inh8}	1.4 s^{-1}	NO degradation	Thomas et al.[11]
k_{inh9}	3.13×10^4 M^{-1}s^{-1}	cGMP activates PKG	*See text below*
k_{inh10}	5×10^{-3} s^{-1}	PKG autoinhibitory deactivation	Inferred from Srihirun et al.[12]
k_{inh11}	1.5×10^{-3} s^{-1}	GMP recycled back to GTP	Inferred from Khan et al.[13] and Mc-kee et al.[14]

In Table S8, the rate constant for NO–sGC binding, k_{inh1a} is based on the intrinsic cellular association rate constant $k_{on} = 1.4$–4.5×10^8 M^{-1}s^{-1}, reported by Sharina et al.[9] Because sGC-expressing cells occupy less volume than the full leaflet, by roughly two orders of magnitude, most NO molecules diffuse without encountering the enzyme. Following the homogenization framework of Nicholson and Bassingthwaighte,[15,16] we scale k_{on} by this volumetric factor to obtain an effective tissue-level rate constant $k_{eff} \approx 0.45$–1.44×10^6 M^{-1}s^{-1}. Based on this, we set $k_{inh1a} = 0.78 \times 10^6$ M^{-1}s^{-1}.

For k_{inh3a}, the rate constant for GTP binding to the NO–sGC complex, we adopted a tissue-level rate corresponding to a cellular value of $\sim 10^5$ M^{-1}s^{-1}. The use of a lower effective rate is intentional: k_{inh3a} is treated as a phenomenological parameter that captures system-level constraints, such as spatial hindrance, allosteric effects, or post-translational modifications, which may reduce the efficiency of GTP association with NO-bound sGC *in vivo*. A similar rationale is used for k_{inh2a}, which describes GTP binding to inactive sGC. Here, we set $k_{inh2a} < k_{inh3a}$, consistent with reports that NO binding induces a conformational change that increases the affinity of sGC for GTP. By encoding this mechanistic asymmetry, rather than assuming equal binding rates,[3] the model more faithfully represents the sequential activation and catalytic behavior of sGC.

For k_{inh9}, although cGMP binds PKG rapidly *in vitro*, we again employ a lower effective rate constant to represent macroscopic factors such as intracellular buffering, compartmentalization, and cooperative activation. The calibrated value yields physiologically reasonable PKG dynamics. For the PKG deactivation rate constant k_{inh10}, we selected a conservative value of 5×10^{-3} s^{-1} based on work by Srihirun et al.,[12] who reported a peak in the PKG activity marker P-VASP Ser239 at approximately 15 min, follwed by a return to baseline by 60 min. Fitting an exponential decay to a 5% residual yields $t_{1/2} \approx 10.4$ min ($k \approx 1.1 \times 10^{-3}$ s^{-1}). However, since the signal may have fully decayed, or simply fallen below the $\sim$ 5% noise floor, we adopt $k = 5 \times 10^{-3}$ s^{-1} to ensure complete deactivation within the observed experimental window.

Finally, the rate constant for GMP $\rightarrow$ GTP recycling, k_{inh11}, was chosen to lie between experimentally grounded lower and upper bounds. A lower bound of $\sim 7.7 \times 10^{-5}$ s^{-1} was inferred from McKee et al.,[14] who observed 13% guanine nucleotide conversion over 30 min in isolated cardiac mitochondria. As an upper bound, we computed an effective first-order rate constant, $k_{inh11}^{upper} = k_{cat}[E]/K_M$, from Michaelis–Menten kinetics (assuming [GMP] $\ll K_M$) using parameters from Figure 6E of Khan et al.[13] and an enzyme concentration of 10 nM, matching their assays. The resulting values spanned 0.015–0.045 s^{-1} across six variants. Because this study characterized only GMP $\rightarrow$ GDP conversion and the assumption [GMP] $\ll K_M$ may fail as GMP

accumulates, the true physiological rate likely falls below this range. Thus, our choice of 1.5×10^{-3} s^{-1} represents a conservative, physiologically plausible estimate.

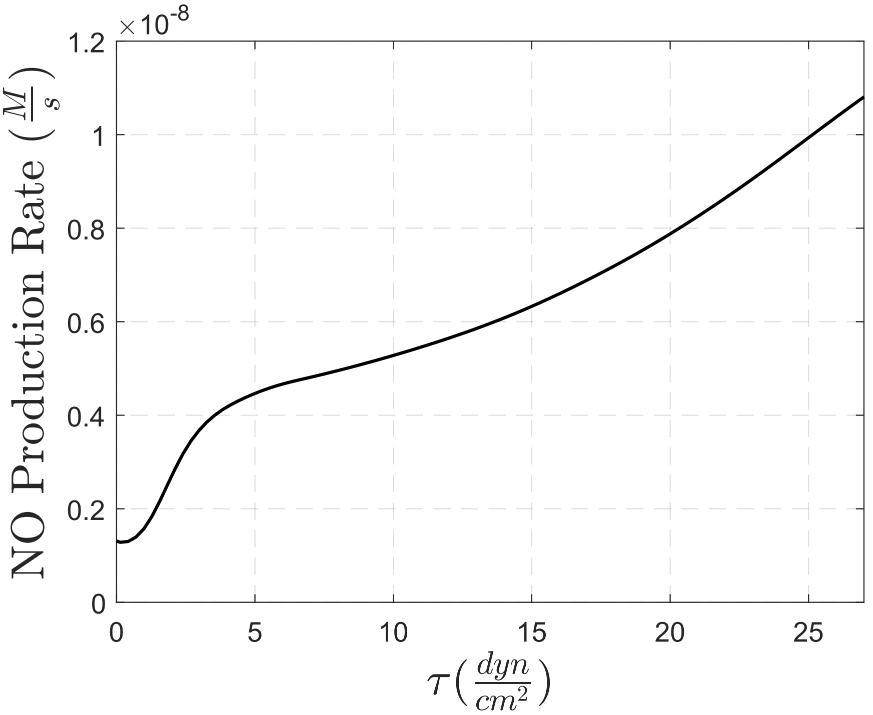

Figure S1: *NO production rate (in M/s) vs. shear stress (in dynes per* cm^2*). Taken from Sriram et al.*[8]

Sensitivity analysis

Method

To quantify the influence of individual model parameters on long-term calcification outcomes, we performed a local, one-at-a-time sensitivity analysis. For a generic model parameter θ, sensitivity was assessed by symmetrically perturbing θ about its nominal value while holding all other parameters fixed. Specifically, parameter values were perturbed as $\theta(1 \pm s)$, and the resulting changes in calcification were evaluated at the terminal simulation time of 23 years.

The sensitivity coefficient was defined as

$$S_\theta = \frac{\mathrm{Ca}(t = 23 \text{ years}; \theta(1+s)) - \mathrm{Ca}(t = 23 \text{ years}; \theta(1-s))}{2\,\mathrm{Ca}(t = 23 \text{ years}; \theta)}, \tag{S50}$$

where $\mathrm{Ca}(t;\theta)$ denotes the model-predicted calcification level at time t for parameter value θ. This normalized metric represents the average relative change in terminal calcification induced by a symmetric perturbation of the parameter and enables direct comparison of sensitivities across parameters with differing units and magnitudes.

All biochemical and kinetic model parameters were perturbed using $s = 0.10$ ($\pm10\%$), consistent with standard local sensitivity analysis practice. For biomechanical inputs, specifically wall shear stress and strain, larger perturbations of $s = 0.15$ ($\pm15\%$) were employed to account for intrinsic cycle-to-cycle variability in these quantities arising from the coupled flow–structure interaction dynamics. Sensitivities were computed independently for each leaflet thickness configuration and reported using the absolute value of S_θ to rank parameter influence on calcification progression.

Stress and strain input sensitivity analysis

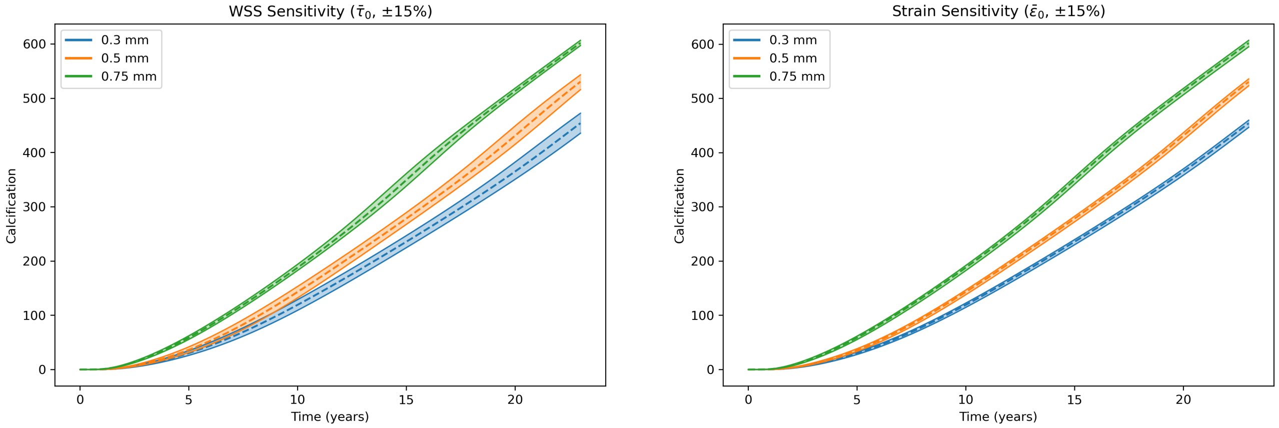

Figure S2: ***Local sensitivity envelopes for biomechanical inputs.*** *Shown are the model-predicted calcification trajectories resulting from symmetric* $\pm 15\%$ *perturbations to the wall shear stress (left) and strain (right) inputs for all three leaflet thickness cases. Shaded regions denote the envelope bounded by the upper and lower perturbation trajectories, while dashed curves indicate the corresponding baseline simulations. Larger perturbations were applied to these biomechanical inputs to account for intrinsic cycle-to-cycle variability arising from the coupled flow–structure interaction dynamics.*

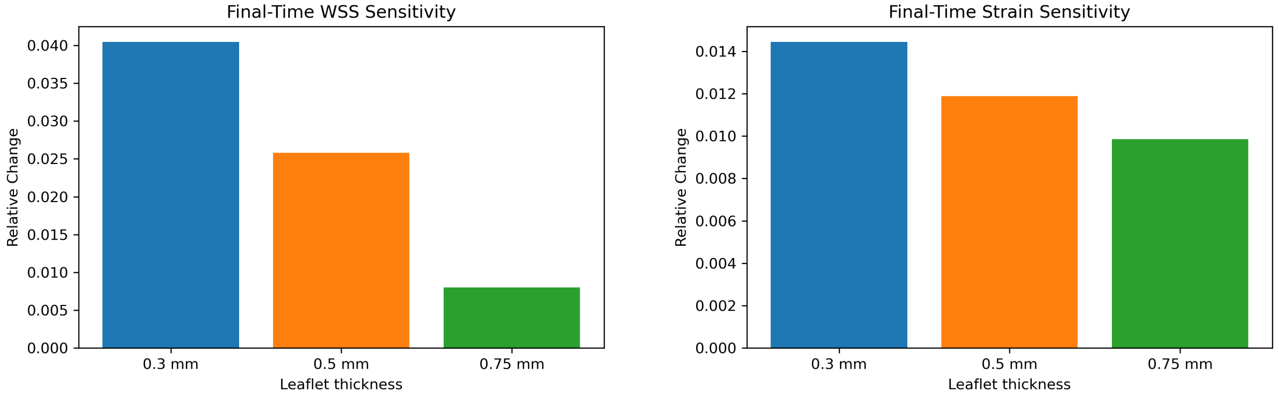

Figure S3: ***Sensitivities of calcification to biomechanical inputs across leaflet thicknesses.*** *Bars show the relative change in terminal calcification at* $t = 23$ *years resulting from symmetric perturbations to wall shear stress (left) and strain (right) for each leaflet thickness case. Sensitivities are computed independently for each input and thickness configuration and quantify the dependence of long-term calcification outcomes on biomechanical loading conditions.*

Model parameter sensitivity analysis

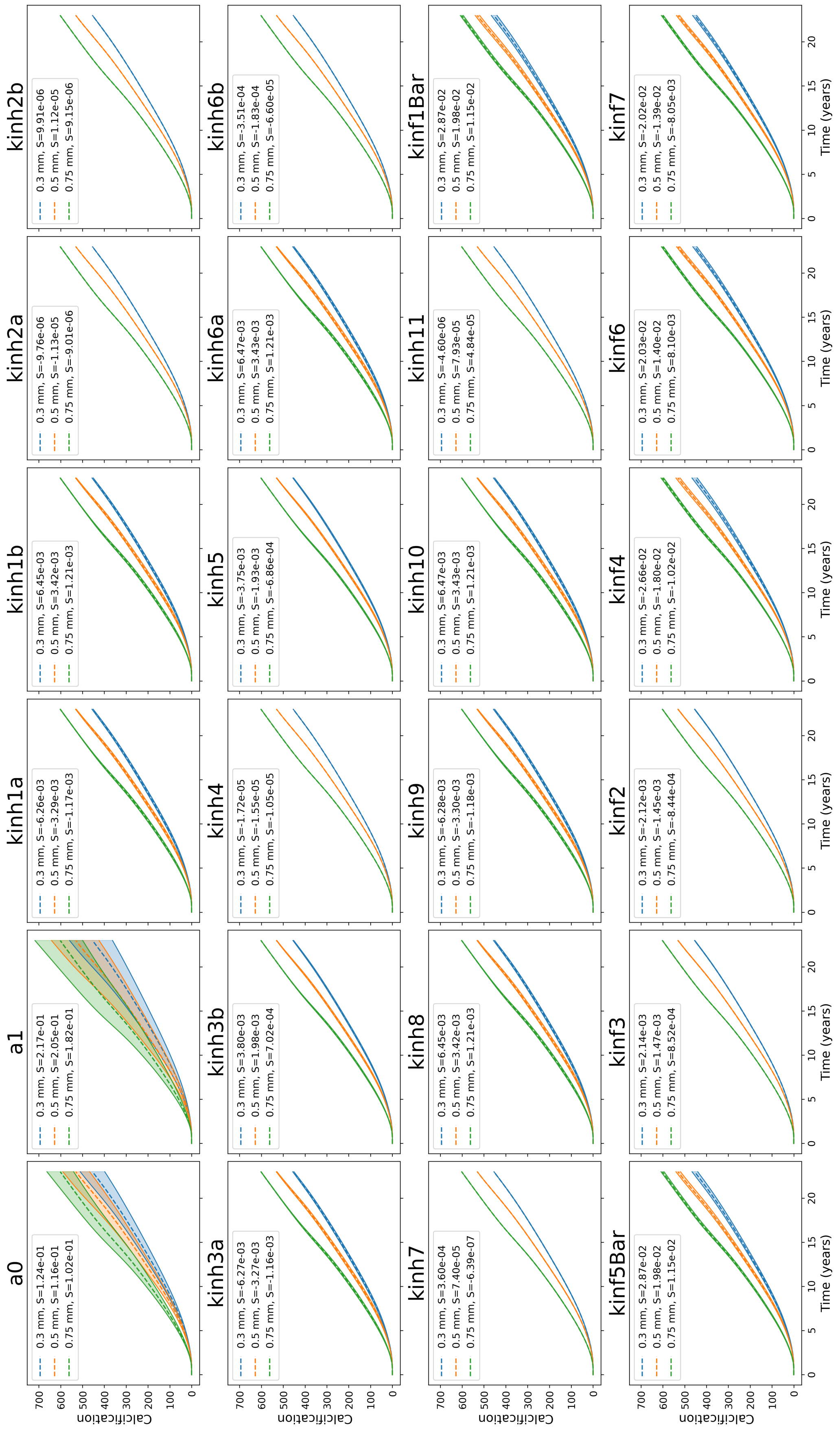

Figure S4: ***Local sensitivity envelopes for all model parameters across leaflet thicknesses.*** *Each panel shows the calcification response to symmetric* $\pm 10\%$ *perturbations for a single parameter, with shaded envelopes corresponding to the three leaflet thickness cases. The dotted curve is the baseline simulation.*

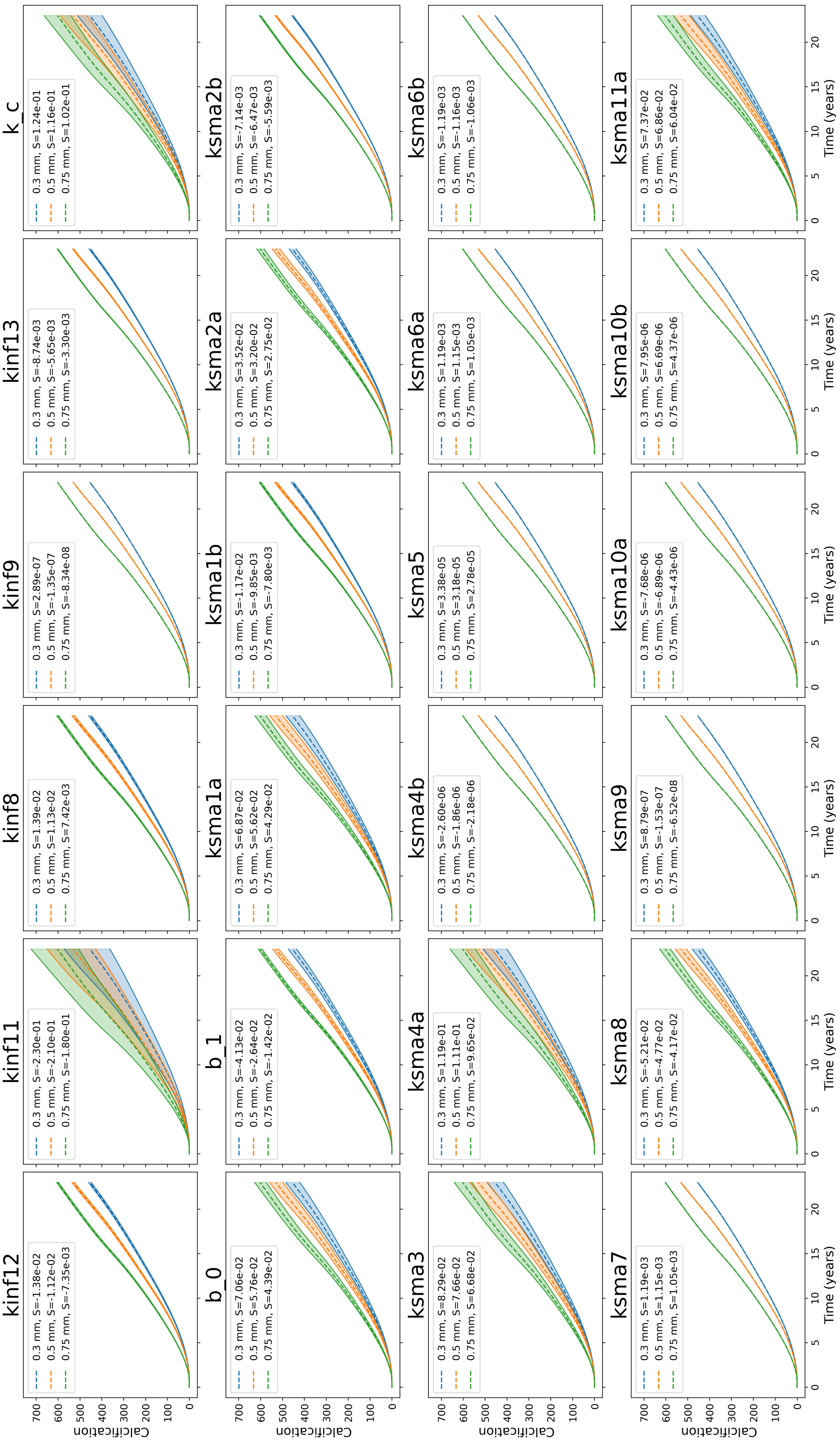

Figure S4: *(continued)*

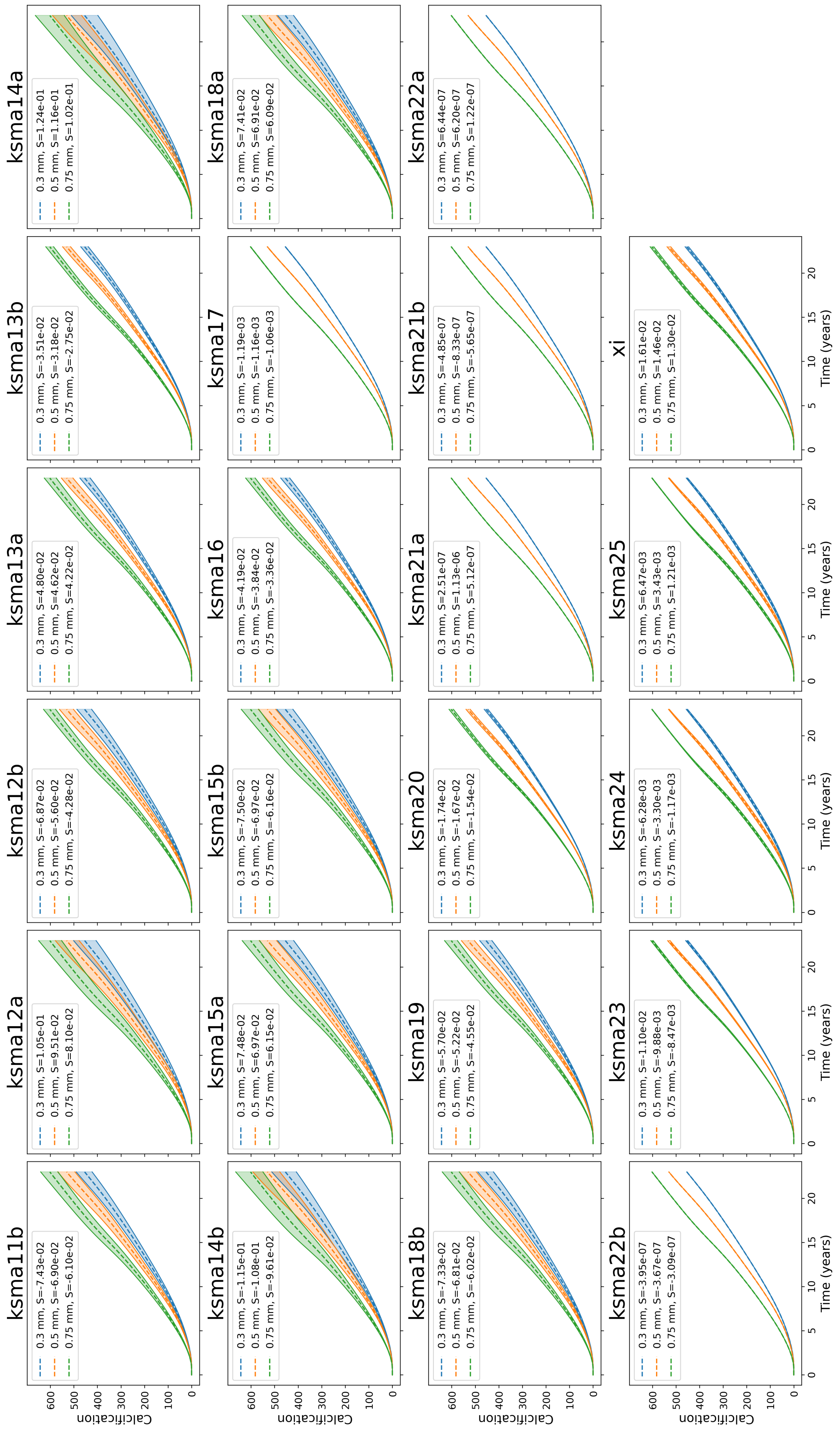

Figure S4: *(continued)*

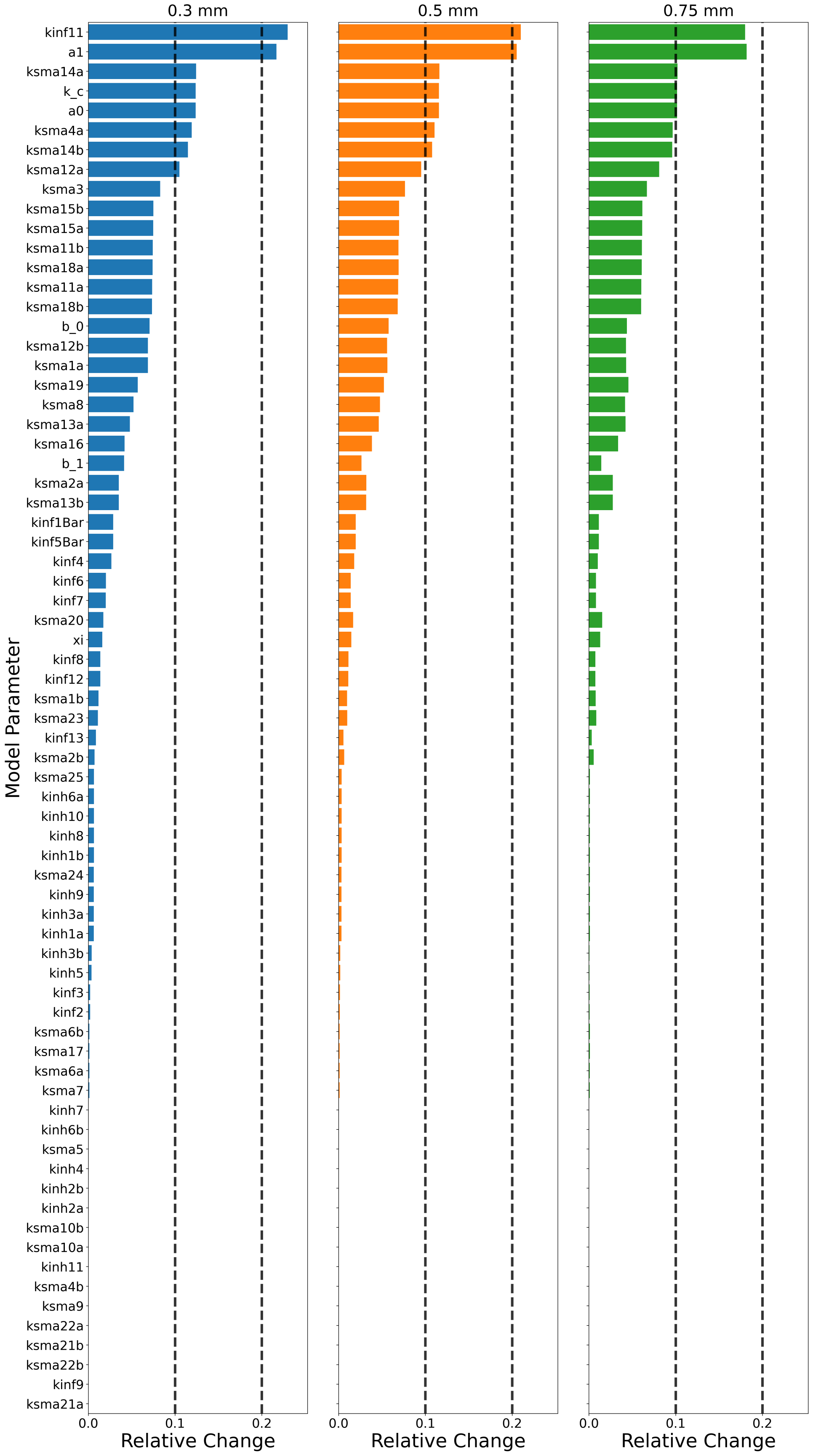

Figure S5: ***Ranked sensitivity of terminal calcification to model parameters.*** *Horizontal bar plots show the relative sensitivity of terminal calcification to individual model parameters for each leaflet thickness case. Parameters are ranked according to sensitivity for the 0.3 mm leaflet, and the same ordering is used for the thicker leaflet cases to facilitate direct comparison. Sensitivity values quantify the relative influence of each parameter on long-term calcification outcomes.*

References

[1] Seung-Wook Chung, Fayth L Miles, Robert A Sikes, Carlton R Cooper, Mary C Farach-Carson, and Babatunde A Ogunnaike. Quantitative modeling and analysis of the transforming growth factor β signaling pathway. *Biophysical Journal*, 96(5):1733–1750, 2009.

[2] Amirhossein Arzani, Kristyn S Masters, and Mohammad RK Mofrad. Multiscale systems biology model of calcific aortic valve disease progression. *ACS Biomaterials Science & Engineering*, 3(11):2922–2933, 2017.

[3] Farshid S Garmaroudi, Diane E Handy, Yang-Yu Liu, and Joseph Loscalzo. Systems pharmacology and rational polypharmacy: nitric oxide-cyclic GMP signaling pathway as an illustrative example and derivation of the general case. *PLoS Computational Biology*, 12(3):e1004822, 2016.

[4] Guo-Wei He and Zhi-Gang Liu. Comparison of nitric oxide release and endothelium-derived hyperpolarizing factor–mediated hyperpolarization between human radial and internal mammary arteries. *Circulation*, 104(suppl_1):I–344, 2001.

[5] Alexandra Keilbach, Peter Ruth, and Franz Hofmann. Detection of cGMP dependent protein kinase isozymes by specific antibodies. *European Journal of Biochemistry*, 208(2):467–473, 1992.

[6] Pei Jiang, Peng-ke YAN, Jian-xiong CHEN, Bing-yang ZHU, Xiao-yong LEI, Wei-dong YIN, and Duan-fang LIAO. High density lipoprotein 3 inhibits oxidized low density lipoprotein-induced apoptosis via promoting cholesterol efflux in RAW264.7 cells. *Acta Pharmacologica Sinica*, 27(2):151–157, 2006.

[7] Geetika Bajpai, Caralin Schneider, Nicole Wong, Andrea Bredemeyer, Maarten Hulsmans, Matthias Nahrendorf, Slava Epelman, Daniel Kreisel, Yongjian Liu, Akinobu Itoh, et al. The human heart contains distinct macrophage subsets with divergent origins and functions. *Nature Medicine*, 24(8):1234–1245, 2018.

[8] Krishna Sriram, Justin G Laughlin, Padmini Rangamani, and Daniel M Tartakovsky. Shear-induced nitric oxide production by endothelial cells. *Biophysical Journal*, 111(1):208–221, 2016.

[9] Iraida Sharina and Emil Martin. Cellular factors that shape the activity or function of nitric oxide-stimulated soluble guanylyl cyclase. *Cells*, 12(3):471, 2023.

[10] Nazish Sayed, Padmamalini Baskaran, Xiaolei Ma, Focco Van Den Akker, and Annie Beuve. Desensitization of soluble guanylyl cyclase, the NO receptor, by S-nitrosylation. *Proceedings of the National Academy of Sciences*, 104(30):12312–12317, 2007.

[11] Douglas D Thomas, Xiaoping Liu, Stephen P Kantrow, and Jack R Lancaster Jr. The biological lifetime of nitric oxide: implications for the perivascular dynamics of NO and O2. *Proceedings of the National Academy of Sciences*, 98(1):355–360, 2001.

[12] Sirada Srihirun, Barbora Piknova, Nathawut Sibmooh, and Alan N Schechter. Phosphorylated vasodilator-stimulated phosphoprotein (P-VASPSer239) in platelets is increased by nitrite and partially deoxygenated erythrocytes. *PLoS One*, 13(3):e0193747, 2018.

[13] Nazimuddin Khan, Parag P Shah, David Ban, Pablo Trigo-Mourino, Marta G Carneiro, Lynn DeLeeuw, William L Dean, John O Trent, Levi J Beverly, Manfred Konrad, et al. Solution structure and functional investigation of human guanylate kinase reveals allosteric networking and a crucial role for the enzyme in cancer. *Journal of Biological Chemistry*, 294(31):11920–11933, 2019.

[14] Edward E McKee, Alice T Bentley, Ronald M Smith Jr, Jonathan R Kraas, and Christina E Ciaccio. Guanine nucleotide transport by atractyloside-sensitive and-insensitive carriers in isolated heart mitochondria. *American Journal of Physiology-Cell Physiology*, 279(6):C1870–C1879, 2000.

[15] Eva Syková and Charles Nicholson. Diffusion in brain extracellular space. *Physiological Reviews*, 88(4):1277–1340, 2008.

[16] JB Bassingthwaighte, CY Wang, and IS3454538 Chan. Blood-tissue exchange via transport and transformation by capillary endothelial cells. *Circulation Research*, 65(4):997–1020, 1989.
